# A3R Phage and *Staphylococcus aureus* Lysate Do Not Induce Neutrophil Degranulation

**DOI:** 10.3390/v9020036

**Published:** 2017-02-21

**Authors:** Jan Borysowski, Ryszard Międzybrodzki, Piotr Wierzbicki, Danuta Kłosowska, Grażyna Korczak-Kowalska, Beata Weber-Dąbrowska, Andrzej Górski

**Affiliations:** 1 Department of Clinical Immunology, Transplantation Institute, Medical University of Warsaw, Nowogrodzka Str. 59, 02-006 Warsaw, Poland; mbrodzki@iitd.pan.wroc.pl (R.M.); selket-anat@wp.pl (P.W.); rovanemi1@wp.pl (D.K.); agorski@ikp.pl (A.G.); 2 Laboratory of Bacteriophages, Ludwik Hirszfeld Institute of Immunology and Experimental Therapy, RudolfaWeigla Str. 12, 53-114 Wrocław, Poland; weber@iitd.pan.wroc.pl; 3 Department of Immunology, University of Warsaw, Miecznikowa Str. 1, 00-001 Warsaw, Poland; korczak-kowalska@wp.pl

**Keywords:** phage, A3R, lysate, phage therapy, neutrophil, granule, degranulation, exocytosis

## Abstract

The objective of this study was to evaluate the effects of A3R phage and *Staphylococcus aureus* lysate obtained after phage infection on neutrophil degranulation. The exocytosis of primary and secondary granules from neutrophils was investigated in vitro in whole blood specimens by flow cytometry based on the expression of specific markers of exocytosis (CD63 for primary granules and CD66b for secondary granules). We found that both A3R and *S. aureus* lysate had no significant effect on the exocytosis of primary and secondary granules. These data suggest that neither A3R virions nor any products of phage-induced lysis of *S. aureus* are likely to induce neutrophil degranulation in patients who are treated with phage preparations. Since neutrophil granules contain some potentially toxic proteins, our results provide an important argument for the safety of phage therapy. Moreover, these data indicate that the induction of neutrophil degranulation is not likely to contribute to antibacterial effects of phages.

## 1. Introduction

While phages are unable to infect eukaryotic cells, many studies have shown that they can interact with some types of these cells, especially with those of the immune system. In fact, phages were shown to influence functions of different types of immune cells involved in the induction of both innate and adaptive immune responses including dendritic cells (DCs), monocytes, neutrophils, as well as T- and B-cells [1,2]. On the other hand, the immune system is the principal factor involved in the clearance of phage virions from blood [3,4]. Thus, research on the interactions between phages and immune cells is important to understand the fate of phage virions in the organism of a patient treated with phage preparations. These studies are also essential for verification of the safety of therapeutic use of phages, since potential activation of immune cells could result in side-effects of phage therapy. For example, excessive activation of some neutrophil functions by phages (or some products of phage-induced lysis of bacterial cells) could cause damage to tissues following the administration of phage preparations. One of the most important functions of neutrophils whose stimulation could cause deleterious effects is degranulation [5].

Neutrophils contain three main granule subsets—primary (azurophilic), secondary (specific), and tertiary that are formed sequentially during terminal granulopoietic differentiation. Individual granule subsets differ from one another by their protein content; their most typical markers include myeloperoxidase (MPO; primary granules), lactoferrin and neutrophil gelatinase-associated lipocalin (NGAL; secondary granules), and gelatinase or matrix metalloproteinase 9 (tertiary granules). Apart from these granules, secretory vesicles are also found in the neutrophil cytoplasm. These are formed by endocytosis at the final stage of granulopoiesis and their proteins are essential for the interactions between neutrophils and endothelial cells [6,7].

It is known that neutrophil degranulation can be induced by some animal and human viruses [8]. Furthermore, a number of studies have shown that the induction of neutrophil degranulation is an important phenomenon occurring in some viral infections [9,10]. Thus, in the present study we examined whether phages can also induce the exocytosis of neutrophil granules. Apart from the effects of phage virions, we investigated whether bacterial lysate obtained after phage infection can induce the exocytosis of neutrophil granules. We included these experiments because, in theory, in patients treated with phage preparations, immunomodulatory effects can be caused not only by phage virions themselves, but also by products of phage-induced lysis of bacteria. The use of a bacterial lysate in in vitro experiments was intended to mimic lysis of bacteria in vivo.

To our knowledge, this is the first study to evaluate the effects of phages and bacterial lysate on neutrophil degranulation.

## 2. Materials and Methods

### 2.1. Phage

A3R phage was obtained from the Therapeutic Phage Collection of Ludwik Hirszfeld Institute of Immunology and Experimental Therapy (IIET), Wrocław, Poland. A3R is a clone of A3 phage which was originally provided by Gerhard Pulverer, Institute of Hygiene, University of Cologne, Germany in 1986, and adapted to therapeutic applications by repeated passaging cycles on clinical *Staphylococcus aureus* strain R19930 (Laboratory of Bacteriophages, IIET). A3R belongs to the Twort-like genus of the *Spounavirinae* subfamily, family *Myoviridae*, order *Caudovirales*. The genome of A3R is composed of double stranded DNA (dsDNA), is 132 kb long, and contains 196 open reading frames (ORFs) ([11]; GenBank Acc. No. JX080301).

A3R was propagated on *S. aureus* strain R19930 (Laboratory of Bacteriophages, IIET). To prepare purified preparation of A3R, crude *S. aureus* lysate obtained after phage infection was subjected to ultrafiltration through polysulfone membranes (Merck Millipore, Kenilworth, NJ, USA) and chromatography on sepharose 4B (Sigma-Aldrich, Poznań, Poland). Stock A3R phage preparations were suspended in phosphate-buffered saline (PBS; Biomed, Lublin, Poland). Phage titer was determined by two-layer method of Adams [12].

### 2.2. Bacterial Lysate

*S. aureus* lysate was prepared by Laboratory of Bacteriophages, IIET, according to the modified method by Slopek [13,14]. In brief, A3R phage was incubated with *S. aureus* strain R19930 in LB medium (Sigma-Aldrich) at 37 °C until complete bacterial lysis occurred (approx. 4–6 h). Next the suspension was filtered through a 0.22-μm filter (Merck Millipore). Stock preparations of lysate were suspended in peptone water (IIET). Phage titer in lysate was measured by two-layer method of Adams [12].

As an additional control for lysate, peptone water (IIET) was used.

### 2.3. Blood Samples

The study was approved by the Ethics Committee of the Medical University of Warsaw (Approval No.: KB/158/2007). Samples of peripheral blood were collected from healthy adult volunteers (*n* = 13) into heparinized tubes.

### 2.4. Neutrophil Degranulation

Neutrophil degranulation was evaluated according to a protocol described by Deree et al. [15] with some modifications. Blood samples (100-µL) were placed into sterile 1.5-mL Eppendorf tubes (Medlab, Raszyn, Poland). The samples were gently mixed and kept at 37 °C in humidified atmosphere (5% CO_2_) for 5 min. Next, 25 µL of A3R phage (titer range 10^6^–10^8^ pfu/mL), *S. aureus* lysate (containing A3R at the titer range 10^6^–10^8^ pfu/mL), PBS (Biomed; the negative control), peptone water (additional control for *S. aureus* lysate), or heat-inactivated *S. aureus* cells suspension (Invitrogen, Waltham, MA, USA; 10^8^ cells/mL; the positive control) were added to individual blood samples. The final experiment volume was adjusted to 200 µL with Hanks' Balanced Salt Solution (HBSS, Biomed). After 20-min incubation at 37 °C in humidified atmosphere (5% CO_2_), samples were placed on ice for 10 min to stop neutrophil degranulation. While on ice, 20 µL of phycoerythrin-conjugated murine anti-human CD63 monoclonal antibody (MAb) (BD Biosciences, San Jose, CA, USA) or 15 µL of fluorescein isothiocyanate (FITC)-conjugated murine anti-human CD66b MAb (BD Biosciences) were added to samples and incubated for 20 min. Optimal volumes of MAbs were determined in preliminary titration experiments. As isotype controls, equal volumes of murine phycoerythrin-conjugated γ1 MAb (BD Biosciences) and murine FITC-conjugated IgM κ isotype MAb (BD Biosciences) were used. Subsequently, erythrocytes were lysed using 1 mL of fluorescence-activated cell sorting (FACS) Lysing Solution (BD Biosciences) for 20 min. Next, samples were centrifuged at 2000 rpm for 5 min, and supernatants were discarded. Cell pellets were washed with PBS with 0.01% sodium azide (Sigma-Aldrich, Saint Louis, MO, USA) at 2000 rpm for 5 min. Supernatants were removed and cell pellets were resuspended in 0.5% formaldehyde solution (Sigma-Aldrich).

The expression of CD63 and CD66b on neutrophils was determined by flow cytometry using FACS Calibur (BD Biosciences) and Cell Quest software. Neutrophil population was identified using forward- and side-scatter plots. A total of 10,000 events was collected for each blood sample. The fluorescence intensity was measured using FLI channel. Separate analyses were performed to determine the percentage of CD63^+^ and CD66^+^ cells in the gated neutrophil population, as well as the mean fluorescence intensity (MFI) of CD63 and CD66b in the gated neutrophil population. Actual values of fluorescence in each experimental variant were calculated by subtracting the fluorescence values measured in cells stained with isotype MAbs from the corresponding values found in cells stained with anti-CD63 MAb or anti-CD66b MAb.

### 2.5. Statistical Analysis

Statistical analysis was performed using Microsoft Excel 2010 and STATISTICA v. 12 software. The results are presented as the mean (*n* = 13) values of analyzed parameters in individual groups ± standard error (SE) of the mean. Statistical differences between individual groups were determined using the Dunn’s test (non-parametric multiple post-hoc comparisons using rank sums). *p* < 0.05 was considered statistically significant.

## 3. Results

To determine the effects of A3R phage and *S. aureus* lysate on neutrophil degranulation, we evaluated the expression of two degranulation markers—CD63 (primary granules) and CD66b (secondary granules). Separate analyses by flow cytometry were performed to determine the percentage of CD63^+^ and CD66b^+^ cells in the gated neutrophil population, as well as the mean fluorescence intensity (MFI) of CD63 and CD66b in the gated neutrophil population.

### 3.1. Effects of A3R Phage on Neutrophil Degranulation

#### 3.1.1. Primary Granules

**Figure 1:**
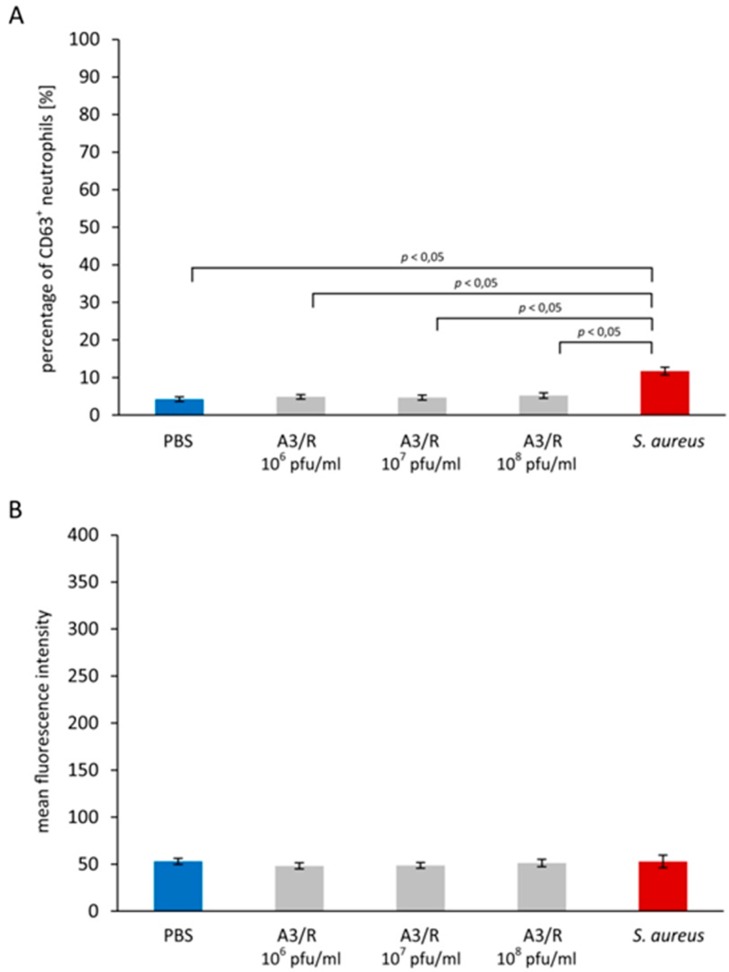

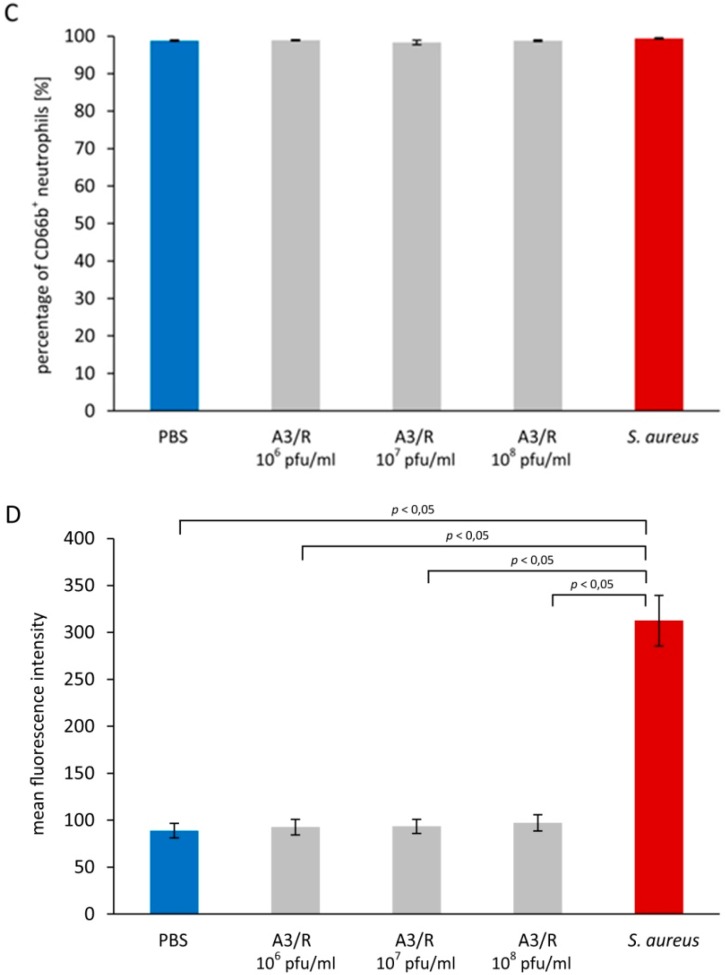
A3R phage does not induce neutrophil degranulation. Neutrophil degranulation was evaluated based on changes of the expression of CD63 (primary granules) and CD66b (secondary granules) on neutrophils. A3R at the titer range 10^6^–10^8^ pfu/mL, phosphate-buffered saline (PBS) (the negative control), or suspension of heat-inactivated *S. aureus* cells (10^8^ cells/mL; the positive control) were added to whole blood samples. After 20-min incubation the percentage of CD63^+^ neutrophils (**A**), the mean fluorescence intensity (MFI) values of CD63 in the gated neutrophil population (**B**), the percentage of CD66b^+^ neutrophils (**C**), and the MFI values of CD66b in the gated neutrophil population (**D**) were determined by flow cytometry. The results shown are the mean percentage values of CD63^+^ and CD66^+^ neutrophils, as well as the MFI values of CD63 and CD66b in the gated neutrophil population ± SE in individual groups. Statistically significant differences (*p* < 0.05) between individual groups are indicated. The experiment was performed on blood samples from 13 blood donors.

We observed no significant difference between the percentage of CD63^+^ neutrophils in blood samples to which the phage was added and the negative control (blood samples to which equal volume of PBS was added); this percentage was 4.86 ± 0.60, 4.69 ± 0.67, 5.21 ± 0.72, and 4.24 ± 0.60 for phage titers 10^6^, 10^7^, 10^8^ pfu/mL, and the control, respectively (Figure 1A).

Unlike A3R, heat-inactivated *S. aureus* cells used as the positive control markedly increased the percentage of CD63^+^ neutrophils. In blood samples to which *S. aureus* cells (10^8^ cells/mL) were added, this percentage was 11.72 ± 1.0 (Figure 1A); the difference between this value and the negative control was statistically significant (*p* = 0.000268). The difference between the percentage of CD63^+^ neutrophils in blood samples to which A3R phage was added and the positive control was also statistically significant for all phage titers (*p* = 0.005882, *p* = 0.001775, and *p* = 0.013122 for titers 10^6^, 10^7^, and 10^8^ pfu/mL, respectively).

We also found that A3R did not significantly increase the MFI of CD63 on neutrophils compared with the negative control; the MFI values were 48.15 ± 3.32, 48.7 ± 3.02, 51.13 ± 3.94, and 52.97 ± 3.26 for phage titers 10^6^, 10^7^, 10^8^ pfu/mL, and the control, respectively (Figure 1B). The MFI of CD63 on neutrophils from blood samples to which heat-inactivated *S. aureus* cells were added was 52.95 ± 6.80 (Figure 1B); there was no significant difference between this value and the negative control. In addition, we did not observe any significant differences between the MFI of CD63 on neutrophils from blood samples to which the phage was added and the positive control.

#### 3.1.2. Secondary Granules

We found no significant differences between the percentage of CD66b^+^ neutrophils in blood samples to which A3R was added and the negative control (98.93 ± 0.13, 98.31 ± 0.61, 98.79 ± 0.68, and 98.8 ± 0.15 for phage titers 10^6^, 10^7^, 10^8^ pfu/mL, and the control, respectively; Figure 1C). In blood samples to which heat-inactivated *S. aureus* cells were added, the percentage of CD66b^+^ neutrophils was also comparable to the negative control (99.38 ± 0.16; Figure 1C).

The MFI values of CD66b on neutrophils from blood samples to which A3R phage was added were comparable with the negative control (92.66 ± 8.22, 93.46 ± 7.48, 97.1 ± 8.63 for phage titers 10^6^, 10^7^, 10^8^ pfu/mL, respectively, and 88.92 ± 7.70 for the control; Figure 1D). *S. aureus* markedly increased the MFI of CD66b on neutrophils to 312.59 ± 27.01 (*p* = 0.000016 compared with the negative control; Figure 1D). The difference between the MFI value found in the positive control and the corresponding MFI values in blood samples to which A3R phage was added was statistically significant for all phage titers (*p* = 0.000104, *p* = 0.000121, and *p* = 0.000222 for titers 10^6^, 10^7^, and 10^8^ pfu/mL, respectively).

### 3.2. Influence of Staphylococcus aureus Lysate on Neutrophil Degranulation

#### 3.2.1. Primary Granules

**Figure 2:**
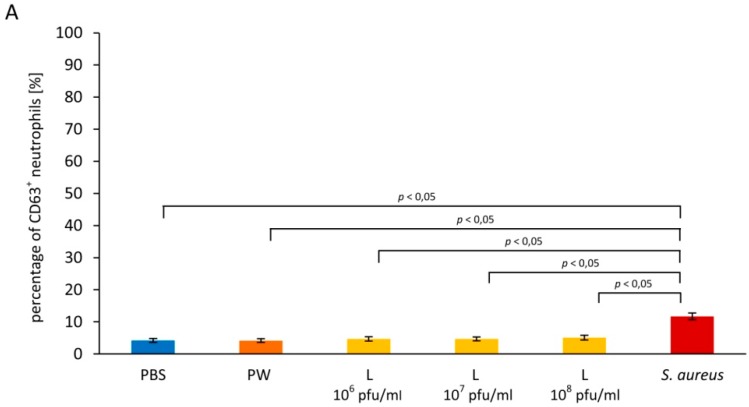

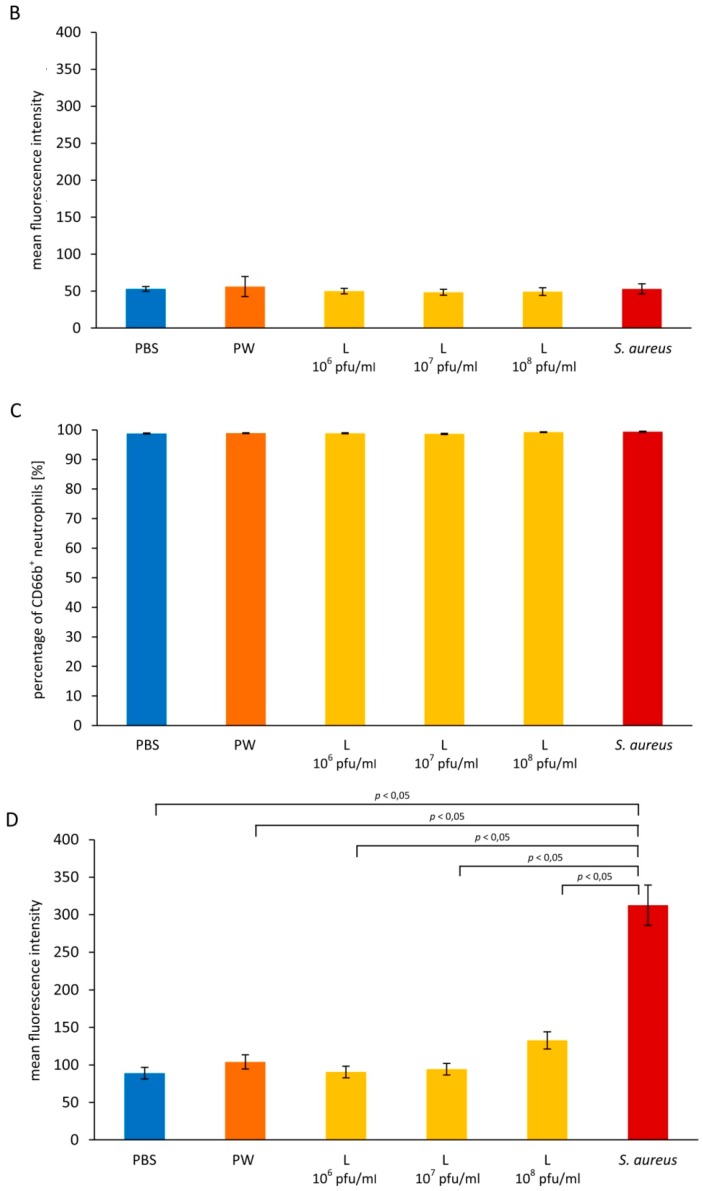
*Staphylococcus aureus* lysate obtained after phage infection has no effect on exocytosis of primary and secondary granules from neutrophils. Exocytosis of primary and secondary granules was evaluated based on changes of the expression of CD63 (primary granules) and CD66b (secondary granules) on neutrophils. *S. aureus* lysate at the titer range 10^6^–10^8^ pfu/mL, PBS (the negative control), peptone water (PW; additional control for lysate), or suspension of heat-inactivated *S. aureus* cells (10^8^ cells/mL; the positive control) were added to whole blood samples. After 20-min incubation the percentage of CD63+ neutrophils (**A**), the mean fluorescence intensity (MFI) values of CD63 in the gated neutrophil population (**B**), the percentage of CD66b+ neutrophils (**C**), and the MFI values of CD66b in the gated neutrophil population (**D**) were determined by flow cytometry. The results shown are the mean percentage values of CD63+ and CD66+ neutrophils, as well as the MFI values of CD63 and CD66b in the gated neutrophil population ± SE in individual groups. Statistically significant differences (*p* < 0.05) between individual groups are indicated. The experiment was performed on blood samples from 13 blood donors.

No significant differences were found between the percentage of CD63^+^ neutrophils in blood samples to which lysate was added and the negative control; the percentage values were 4.68 ± 0.68, 4.68 ± 0.60, 5.08 ± 0.73 for lysate titers 10^6^, 10^7^, 10^8^ pfu/mL, respectively, and 4.25 ± 0.60 for the control (Figure 2A). The difference between the percentage of CD63^+^ neutrophils in blood samples to which lysate was added and the positive control (11.72 ± 1.0) was statistically significant for all lysate titers (*p* = 0.001607, *p* = 0.003037, and *p* = 0.008110 for titers 10^6^, 10^7^, and 10^8^ pfu/mL, respectively).

The MFI values of CD63 on neutrophils were comparable in blood samples to which lysate was added and the negative control (49.95 ± 3.67, 48.33 ± 4.07, and 49.24 ± 5.16 for titers 10^6^, 10^7^, and 10^8^ pfu/mL, respectively, and 52.97 ± 3.26 for the control; Figure 2B). We also found no significant differences between the MFI of CD63 on neutrophils in blood samples to which lysate was added and the positive control (52.95 ± 6.80; *p* > 0.05).

#### 3.2.2. Secondary Granules

We noted no significant differences between the percentage of CD66b^+^ neutrophils in blood samples to which lysate was added (98.85 ± 0.17, 98.64 ± 0.21, 99.23 ± 0.11 for titers 10^6^, 10^7^, and 10^8^ pfu/mL, respectively) and the negative control (98.8 ± 0.15; Figure 2C). The difference between the percentage of CD66b^+^ neutrophils in blood samples to which lysate was added and the positive control (99.38 ± 0.16) was also not statistically significant (*p* > 0.05).

The MFI values of CD66b on neutrophils were comparable in blood samples to which lysate was added and the negative control (90.47 ± 7.76, 94.28 ± 7.82, 132.75 ± 11.54, and 88.92 ± 7.70 for titers 10^6^, 10^7^, 10^8^ pfu/mL, and for the control, respectively; Figure 2D). In the positive control, the MFI of CD66b was 312.59 ± 27.01. The difference between the MFI value of CD66b on neutrophils from blood samples to which lysate was added and the positive control was statistically significant for the low and the medium lysate titer (*p* = 0.000028 and *p* = 0.000130, respectively); for blood samples to which lysate at the high titer was added, no significant difference with the positive control was found.

## 4. Discussion

The main objective of this study was to evaluate whether phages induce neutrophil degranulation. As a model bacterial virus, we employed an obligatory lytic, polyvalent staphylococcal A3R phage that was described recently [11].

Theoretically, the exocytosis of neutrophil granules might be induced not only by A3R virions, but also by some products of phage-induced lysis of *S. aureus* cells (likely to occur during the treatment of a bacterial infection with a phage preparation). Therefore, apart from A3R virions, in our experiments we also used *S. aureus* lysate obtained after phage infection. The use of *S. aureus* lysate in in vitro experiments was intended to mimic lysis of bacteria in vivo. While lysates also contain phage particles, potential immunomodulatory effects of these particles could be reduced as a result of binding to some components of bacterial cells. It also needs to be stressed that currently lysates rather than purified phages are used in the treatment of bacterial infections [16]. However, since purified phages can also be used in the treatment of bacterial infections in the future, performing separate experiments with purified phage virions and lysates seemed warranted (only this way can firm evidence of safety of both kinds of preparations be obtained).

It is known that individual granule subsets differ in their capacity for exocytosis as a result of neutrophil activation [7]. In this study, we focused on the exocytosis of two main kinds of neutrophil granules—primary and secondary granules. Primary granules contain most proteins that can be potentially toxic for human tissues, and they are mobilized relatively less readily compared with other kinds of granules [17]. Thus, by investigating secretion of the contents of primary and secondary granules, we wanted to determine whether A3R and *S. aureus* lysate can induce the exocytosis of granules that are easy and difficult to mobilize from the cytoplasm.

We found that neither A3R nor *S. aureus* lysate significantly induced the exocytosis of primary and secondary granules. Both the percentages of CD63^+^ and CD66b^+^ neutrophils, as well as the MFI values of CD63 and CD66b on neutrophils, were comparable in blood samples to which either of the studied preparations was added and the negative control.

Research into the influence of phages on neutrophil degranulation is especially important in view of the results of studies that showed that some animal and human viruses can induce the exocytosis of neutrophil granules. Some proteins from these granules have been implicated in the pathogenesis of viral infections. For example, in children infected with dengue virus a higher concentration of elastase was found in the peripheral blood compared with healthy children [10]. A higher concentration of elastase was also reported in sputum of patients with viral exacerbation of asthma compared with patients with uncomplicated asthma; neutrophil activation, including the induction of granule exocytosis, is associated with a more severe clinical course of asthma and longer hospital stays of patients [18]. Moreover, a higher level of spontaneous degranulation was found in neutrophils isolated from patients with human immunodeficiency virus (HIV) infections compared with healthy individuals [19]. Furthermore, higher concentrations of some degranulation markers, including MPO, arginase I, and NGAL were reported in sera of patients with HIV infections; some of these proteins can contribute to T cell dysfunction, a hallmark of acquired immune deficiency syndrome (AIDS) [9]. There are also reports on neutrophil degranulation induced by type A influenza virus [20] and respiratory syncytial virus (RSV) [8].

In the context of the above data, our results provide an important argument for the safety of therapeutic applications of phages. These results suggest that, unlike animal and human viruses, A3R phage, as well as the products of A3R-caused lysis of *S. aureus* (likely to occur in the treated patients) should not cause tissue damage in patients as a result of the release of potentially toxic mediators from neutrophil granules. Whether or not other phages can induce neutrophil degranulation should be verified in future studies. In particular, it needs to be investigated whether the exocytosis of neutrophil granules can be induced by lysates of Gram-negative bacteria containing other potentially immunoreactive components (especially lipopolysaccharides, LPS).

An interesting phenomenon is the induction of neutrophil degranulation by intact *S. aureus* cells used as the positive control coupled with a lack of any effect of *S. aureus* lysate on the exocytosis of granules. These results are in line with the findings from our previous study showing that *S. aureus* lysate obtained after phage infection had no effect on the production of reactive oxygen species in monocytes and neutrophils, while intact heat-inactivated *S. aureus* cells induced a very strong respiratory burst in both monocytes and neutrophils [21]. While different strains of *S. aureus* were used as the positive control and as the host for A3R propagation in the present study, this is likely not the reason for the observed discrepancy. Rather, we believe that A3R phage virions present in *S. aureus* lysate can bind to some of the potentially immunoreactive components of bacterial cells to neutralize them. This hypothesis derives some indirect experimental support from the results of our previous study demonstrating that as a result of binding to LPS molecules T4 phage can reduce the intensity of the LPS-induced respiratory burst by neutrophils [22]. Thus, it is possible that A3R phage virions can also bind to some components of *S. aureus* cells present in lysate which otherwise would induce neutrophil degranulation. Regretfully, the receptors for A3R phage have not been identified yet. Furthermore, to verify our hypothesis, further studies should be performed to evaluate whether *S. aureus* lysate that does not contain phage virions can induce neutrophil degranulation. Were our hypothesis true, this would imply that phage preparations may not only directly eliminate susceptible bacteria, but also prevent excessive activation of some neutrophil functions (especially degranulation or the respiratory burst) which otherwise could adversely affect tissues of patients with bacterial infections.

In our previous studies, we showed that A3R and T4 phages as well as *S. aureus* and *Escherichia coli* lysates, unlike animal and human viruses and intact bacterial cells, did not induce a significant respiratory burst in neutrophils and monocytes in vitro [21,23]. Moreover, we found that T4 can inhibit both bacteria- and LPS-induced respiratory burst in neutrophils [22]. These results suggest that phage preparations are not likely to cause oxidative stress in tissues of the treated patients as a result of an excessive stimulation of the respiratory burst. Thus, based on our previous and current results, we conclude that phages are not likely to exert any deleterious neutrophil-mediated effects in patients treated with phage preparations.

## References

[1] An T.W., Kim S.J., Lee Y.D., Park J.H., Chang H.I. (2014). The immune-enhancing effect of the *Cronobacter sakazakii* ES2 phage results in the activation of nuclear factor-κB and dendritic cell maturation via the activation of IL-12p40 in the mouse bone marrow. Immunol. Lett..

[2] Górski A., Międzybrodzki R., Borysowski J., Dąbrowska K., Wierzbicki P., Ohams M., Korczak-Kowalska G., Olszowska-Zaremba N., Łusiak-Szelachowska M. (2012). Phage as a modulator of immune responses: Practical implications for phage therapy. Adv. Virus Res..

[3] Hodyra-Stefaniak K., Miernikiewicz P., Drapała J., Drab M., Jończyk-Matysiak E., Lecion D., Kaźmierczak Z., Beta W., Majewska J., Harhala M. (2015). Mammalian Host-Versus-Phage immune response determines phage fate in vivo. Sci. Rep..

[4] Srivastava A.S., Kaido T., Carrier E. (2004). Immunological factors that affect the in vivo fate of T7 phage in the mouse. J. Virol. Methods.

[5] Boudiaf K., Hurtado-Nedelec M., Belambri S.A., Marie J.C., Derradji Y., Benboubetra M., El-Benna J., Dang P.M. (2016). Thymoquinone strongly inhibits fMLF-induced neutrophil functions and exhibits anti-inflammatory properties in vivo. Biochem. Pharmacol..

[6] Sheshachalam A., Srivastava N., Mitchell T., Lacy P., Eitzen G. (2014). Granule protein processing and regulated secretion in neutrophils. Front. Immunol..

[7] Rørvig S., Østergaard O., Heegaard N.H., Borregaard N. (2013). Proteome profiling of human neutrophil granule subsets, secretory vesicles, and cell membrane: Correlation with transcriptome profiling of neutrophil precursors. J. Leukoc. Biol..

[8] Jaovisidha P., Peeples M.E., Brees A.A., Carpenter L.R., Moy J.N. (1999). Respiratory syncytial virus stimulates neutrophil degranulation and chemokine release. J. Immunol..

[9] Bowers N.L., Helton E.S., Huijbregts R.P., Goepfert P.A., Heath S.L., Hel Z. (2014). Immune suppression by neutrophils in HIV-1 infection: Role of PD-L1/PD-1 pathway. PLoS Pathog..

[10] Juffrie M., van Der Meer G.M., Hack C.E., Haasnoot K., Sutaryo, Veerman A.J., Thijs L.G. (2000). Inflammatory mediators in dengue virus infection in children: Interleukin-8 and its relationship to neutrophil degranulation. Infect. Immun..

[11] Łobocka M., Hejnowicz M.S., Dąbrowski K., Gozdek A., Kosakowski J., Witkowska M., Ulatowska M.I., Weber-Dąbrowska B., Kwiatek M., Parasion S. (2012). Genomics of staphylococcal Twort-like phages—Potential therapeutics of the post-antibiotic era. Adv. Virus Res..

[12] Adams M.H. (1959). Bacteriophages.

[13] Slopek S., Durlakowa I., Weber-Dabrowska B., Kucharewicz-Krukowska A., Dabrowski M., Bisikiewicz R. (1983). Results of bacteriophage treatment of suppurative bacterial infections. I. General evaluation of the results. Arch. Immunol. Ther. Exp..

[14] Letkiewicz S., Miedzybrodzki R., Fortuna W., Weber-Dabrowska B., Górski A. (2009). Eradication of *Enterococcus faecalis* by phage therapy in chronic bacterial prostatitis—Case report. Folia Microbiol..

[15] Deree J., Lall R., Melbostad H., Grant M., Hoyt D.B., Coimbra R. (2006). Neutrophil degranulation and the effects of phosphodiesterase inhibition. J. Surg. Res..

[16] Międzybrodzki R., Borysowski J., Weber-Dąbrowska B., Fortuna W., Letkiewicz S., Szufnarowski K., Pawełczyk Z., Rogóż P., Kłak M., Wojtasik E. (2012). Clinical aspects of phage therapy. Adv. Virus Res..

[17] Mollinedo F., Martín-Martín B., Calafat J., Nabokina S.M., Lazo P.A. (2003). Role of vesicle-associated membrane protein-2, through Q-soluble N-ethylmaleimide-sensitive factor attachment protein receptor/R-soluble N-ethylmaleimide-sensitive factor attachment protein receptor interaction, in the exocytosis of specific and tertiary granules of human neutrophils. J. Immunol..

[18] Wark P.A., Johnston S.L., Moric I., Simpson J.L., Hensley M.J., Gibson P.G. (2002). Neutrophil degranulation and cell lysis is associated with clinical severity in virus-induced asthma. Eur. Respir. J..

[19] Meddows-Taylor S., Pendle S., Tiemessen C.T. (2001). Altered expression of CD88 and associated impairment of complement 5a-induced neutrophil responses in human immunodeficiency virus type 1-infected patients with and without pulmonary tuberculosis. J. Infect. Dis..

[20] Henricks P.A., van der Tol M.E., Verhoef J. (1985). Interactions between human polymorphonuclear leukocytes and influenza virus. Scand. J. Immunol..

[21] Borysowski J., Wierzbicki P., Kłosowska D., Korczak-Kowalska G., Weber-Dabrowska B., Górski A. (2010). The effects of T4 and A3/R phage preparations on whole-blood monocyte and neutrophil respiratory burst. Viral. Immunol..

[22] Międzybrodzki R., Switala-Jelen K., Fortuna W., Weber-Dabrowska B., Przerwa A., Lusiak-Szelachowska M., Dabrowska K., Kurzepa A., Boratynski J., Syper D. (2008). Bacteriophage preparation inhibition of reactive oxygen species generation by endotoxin-stimulated polymorphonuclear leukocytes. Virus Res..

[23] Przerwa A., Zimecki M., Switała-Jeleń K., Dabrowska K., Krawczyk E., Łuczak M., Weber-Dabrowska B., Syper D., Międzybrodzki R., Górski A. (2006). Effects of bacteriophages on free radical production and phagocytic functions. Med. Microbiol. Immunol..

